# Genomic Surveillance of Respiratory Pathogens: From Molecular Detection to Precision Infection Control

**DOI:** 10.3390/pathogens15101044

**Published:** 2026-10-02

**Authors:** Jiahui Chen, Qihui Zou, Benshan Pan, Yifei Zhang

**Affiliations:** 1 Sanya Institute of Hunan University of Science and Technology, Sanya 572024, China; jiahuichen994@gmail.com (J.C.); z18273387603@163.com (Q.Z.); pbs200304@163.com (B.P.); 2 School of Life and Health Sciences, Hunan University of Science and Technology, Xiangtan 411201, China

**Keywords:** respiratory pathogens, genomic surveillance, molecular diagnostics, whole-genome sequencing, metagenomic sequencing, artificial intelligence, public health surveillance

## Abstract

Respiratory pathogens remain a major global public health concern due to factors including their genetic diversity, dynamic transmission patterns, and increasing antimicrobial resistance. Conventional molecular diagnostics, particularly polymerase chain reaction (PCR)-based assays, remain essential for rapid and scalable pathogen detection but provide limited information on pathogen evolution, transmission, and resistance mechanisms. Advances in whole-genome sequencing (WGS), targeted sequencing, and metagenomic next-generation sequencing (mNGS) have expanded respiratory pathogen surveillance from targeted detection toward higher-resolution genomic characterization. However, no single technology can adequately address all surveillance objectives. Instead, molecular diagnostics, genomic sequencing, epidemiological investigation, and clinical assessment provide complementary information that can be integrated according to the pathogen, clinical context, and specific public health question. This review examines the evolution of respiratory pathogen surveillance and emphasizes an integrated framework in which different technologies are selected and combined to support pathogen characterization, transmission investigation, antimicrobial resistance surveillance, and preventive decision-making. We further discuss emerging applications of artificial intelligence (AI) for integrating genomic, epidemiological, and clinical data, with potential roles in genomic interpretation, early risk assessment, and precision infection control. Although AI-enhanced surveillance remains largely at the research and validation stage, advances in data integration, model development, and computational infrastructure may enable more predictive and actionable surveillance systems. Overall, the future of respiratory pathogen surveillance will depend not simply on adopting more advanced technologies, but on integrating complementary detection and analytical approaches to transform pathogen detection into actionable evidence for public health decision-making.

## 1. Introduction

### 1.1. Respiratory Pathogens: A Growing Surveillance Challenge

Respiratory pathogens remain a leading cause of infectious disease morbidity and mortality worldwide, posing substantial challenges to healthcare systems and public health authorities [1,2]. Seasonal influenza viruses, respiratory syncytial virus (RSV), severe acute respiratory syndrome coronavirus 2 (SARS-CoV-2), adenoviruses, and important bacterial pathogens such as *Klebsiella pneumoniae* and *Streptococcus pneumoniae* collectively account for a considerable proportion of respiratory infections across all age groups [3]. The COVID-19 pandemic further demonstrated that respiratory pathogens can rapidly evolve from localized outbreaks into global public health emergencies, emphasizing the critical importance of effective surveillance systems capable of supporting timely detection and response [4,5].

The increasing complexity of respiratory pathogens has made surveillance considerably more challenging than simple pathogen detection. Many respiratory viruses exhibit high mutation rates [6,7], allowing continuous genetic diversification that may alter transmissibility, virulence, immune escape [8], and vaccine effectiveness. Meanwhile, multiple viral and bacterial pathogens frequently co-circulate during respiratory infection seasons and often present with highly overlapping clinical manifestations [9,10], making etiological diagnosis and epidemiological investigation increasingly difficult. In addition, the growing burden of antimicrobial-resistant bacterial pathogens further complicates clinical management and infection prevention [11]. Together, these characteristics have expanded the objectives of surveillance from identifying pathogens to understanding how they evolve, spread, and respond to interventions.

Conventional laboratory methods, including culture, PCR, qPCR, and multiplex molecular assays, remain essential for respiratory pathogen detection. However, these approaches generally provide limited information on pathogen evolution, transmission dynamics, and resistance mechanisms. As surveillance objectives have expanded beyond detection, complementary genomic and epidemiological approaches are increasingly required to provide higher-resolution information for public health decision-making.

However, access to genomic sequencing and the integration of genomic data into public health surveillance remain uneven across settings, particularly where laboratory infrastructure and technical capacity are limited. Strengthening genomic capacity and translating genomic information into actionable public health responses remain important priorities [12,13].

### 1.2. From Molecular Detection to Integrated Genomic Surveillance

Over the past decades, respiratory pathogen surveillance has progressively expanded from confirming the presence of known pathogens to generating higher-resolution information on pathogen evolution, transmission, and antimicrobial resistance [14,15,16,17]. This shift has increased the need for surveillance systems that can integrate laboratory findings with genomic and epidemiological evidence to support public health decision-making.

The practical value of genomic approaches became particularly evident during major infectious disease events. In the 2011 outbreak of carbapenem-resistant *Klebsiella pneumoniae* at the U.S. National Institutes of Health Clinical Center, whole-genome sequencing (WGS) was integrated with epidemiological information to investigate an outbreak involving 18 patients [18]. Genomic and epidemiological analyses identified three independent transmission events originating from a single patient who had been discharged before subsequent cases became clinically apparent, providing evidence that was difficult to obtain from conventional epidemiological investigation alone. This study demonstrated how high-resolution genomic information could complement routine epidemiological investigation and improve the reconstruction of healthcare-associated transmission pathways.

The potential of genomic approaches for identifying previously unrecognized respiratory pathogens was demonstrated during the emergence of the novel coronavirus in Wuhan in late 2019. Metagenomic sequencing of bronchoalveolar lavage fluid from a patient with unexplained pneumonia identified a previously unrecognized coronavirus, and subsequent genome assembly and targeted PCR generated a near-complete genome sequence that enabled rapid characterization of the virus [19]. Independent studies subsequently obtained full-length genome sequences from additional patients at an early stage of the outbreak. These findings provided a critical molecular foundation for diagnostic assay development, epidemiological investigation, and subsequent genomic surveillance of the emerging virus.

Recent advances in genomic sequencing technologies have expanded respiratory pathogen surveillance beyond targeted molecular detection. Whole-genome sequencing (WGS), metagenomic next-generation sequencing (mNGS), and emerging rapid sequencing approaches can provide complementary genomic information for pathogen characterization, outbreak investigation, and public health surveillance [20,21]. The World Health Organization (WHO, Geneva, Switzerland) Global Genomic Surveillance Strategy similarly emphasizes an end-to-end framework linking diagnostics, sequencing, bioinformatics, data sharing, and public health action [22,23].

**Figure 1:**
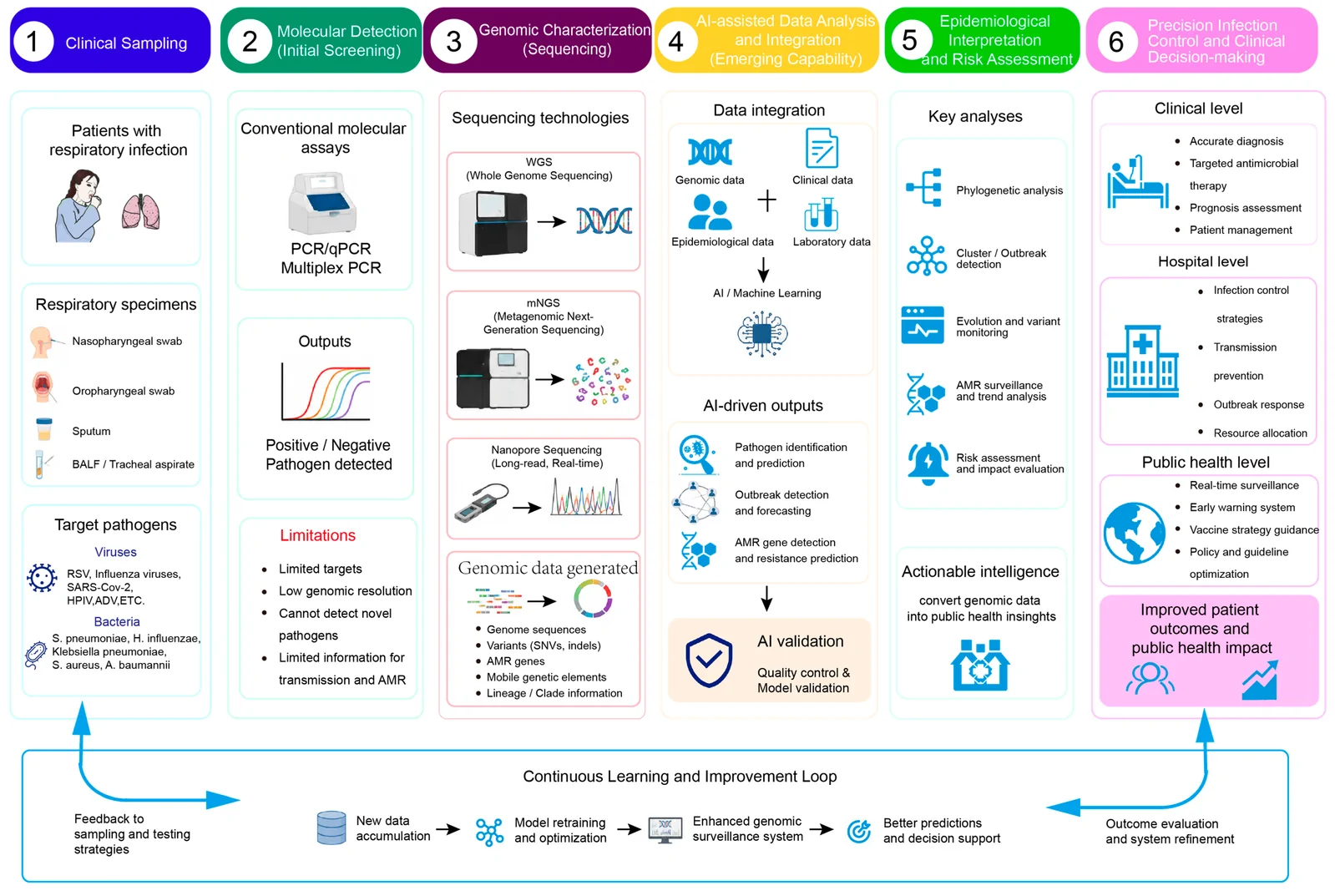
Conceptual Framework of Genomic Surveillance for Respiratory Pathogens: From Clinical Samples to Precision Infection Control. Image adapted from Servier Medical Art (https://smart.servier.com/ (accessed on 5 August 2026)), licensed under CC BY 4.0.

Against this background, integrated genomic surveillance provides a framework for combining molecular diagnostics, genomic sequencing, epidemiological investigation, and data analysis according to specific surveillance objectives. This review examines the technological development and public health applications of respiratory pathogen genomic surveillance, with emphasis on how complementary approaches can support pathogen characterization, transmission investigation, antimicrobial resistance surveillance, and precision infection control. We also discuss emerging AI applications and the practical challenges of translating these approaches into routine surveillance (Figure 1).

## 2. Molecular Detection: The Foundation of Respiratory Pathogen Surveillance

Molecular diagnostics remain a foundation of respiratory pathogen surveillance because they enable rapid and scalable detection and provide the laboratory data needed for subsequent epidemiological and genomic analysis [24].

### 2.1. Conventional Molecular Diagnostics

Polymerase chain reaction (PCR) and quantitative PCR (qPCR/RT-qPCR) remain fundamental tools for respiratory pathogen detection because of their rapid turnaround time, high analytical sensitivity, and established laboratory workflows [25]. During the COVID-19 pandemic, RT-qPCR supported large-scale detection of SARS-CoV-2 and provided laboratory-confirmed data for epidemiological surveillance and outbreak response. Similar molecular assays remain central to routine surveillance of influenza viruses, respiratory syncytial virus (RSV), and other respiratory pathogens, and the World Health Organization (WHO) continues to incorporate molecular testing into integrated respiratory virus surveillance [26,27].

The surveillance value of molecular diagnostics extends beyond individual diagnosis. Repeated molecular testing can provides standardized indicators of pathogen activity, positivity trends, and seasonal circulation. For example, WHO’s Global Influenza Surveillance and Response System (GISRS) integrates laboratory detection with epidemiological information through platforms such as FluNet and RespiMart, enabling respiratory virus activity to be monitored across countries and over time [28,29]. However, conventional PCR assays generally target predefined genomic regions, their surveillance value is primarily detection-oriented rather than genome-resolving. They can establish whether a pathogen or specific target is present, but usually provide limited information on lineage assignment, genome-wide variation, or fine-scale transmission relationships [30]. These limitations provide a rationale for incorporating multiplex and genomic approaches into broader surveillance frameworks.

### 2.2. Multiplex Molecular Detection

The limitations of single-target testing have encouraged the adoption of multiplex molecular panels, particularly in clinical settings where multiple respiratory pathogens may produce overlapping syndromes [26]. Multiplex PCR allows several predefined viral and bacterial targets to be assessed from a single specimen and can therefore increase the information obtained from each clinical encounter [31].

Clinical studies have demonstrated that multiplex molecular testing can improve diagnostic yield and shorten reporting time compared with conventional batch-based testing [32]. In a prospective study of adults with respiratory illness in Australia, the FilmArray respiratory panel reduced the median turnaround time from 21.6 to 4.4 h and increased the diagnostic yield from 39.5% to 57.3% compared with in-house batch PCR [33]. Consistent with these findings, a systematic review including 20 studies and 5510 respiratory specimens further reported high overall diagnostic accuracy for multiplex PCR, with AUROC values ≥0.98 for most evaluated respiratory viruses [34]. However, these improvements in diagnostic performance do not necessarily translate directly into improved population-level surveillance performance. When integrated into systematic sampling and surveillance networks, multiplex testing may nevertheless enhance surveillance sensitivity and timeliness by enabling more rapid and comprehensive detection of circulating pathogens.

At the surveillance level, multiplex panels remain constrained by their predefined target composition. Nevertheless, targeted RT-PCR and multiplex assays are incorporated into WHO respiratory virus surveillance algorithms for the detection and typing of RSV and influenza according to defined surveillance objectives [35]. Their principal value is therefore rapid and broad frontline detection, while genomic approaches provide higher-resolution information for monitoring pathogen evolution, reconstructing transmission patterns, and characterizing genetic variation when epidemiologically or clinically indicated (Table 1).

### 2.3. From Targeted Molecular Detection to Genomic Surveillance

Targeted molecular diagnostics have become indispensable for routine detection of respiratory pathogens; however, their surveillance value is inherently constrained by predefined targets. PCR-based assays can rapidly establish pathogen presence, but provide limited information on genetic diversity, evolutionary relationships, transmission patterns, and the broader spectrum of antimicrobial resistance determinants. Moreover, pathogen evolution may alter the performance or interpretability of targeted assays.

The emergence of SARS-CoV-2 variants illustrates this limitation. During the emergence of the Alpha variant in the United Kingdom in late 2020, routine RT-qPCR could confirm SARS-CoV-2 infection but could not directly determine viral lineage. Although S-gene target failure (SGTF) subsequently provided a useful proxy for preliminary screening of Alpha, definitive variant identification still required genomic sequencing [42]. Similar challenges have been observed for other respiratory pathogens: genetic changes in circulating A(H3N2) viruses have been reported to affect molecular assay performance, while targeted detection of carbapenemase genes may miss less common resistance variants such as OXA-181 [8,43,44]. These examples highlight the dependence of targeted surveillance on predefined genetic targets and their continuous adaptation to pathogen evolution [45].

As respiratory pathogen surveillance increasingly supports outbreak investigation and public health decision-making, these limitations have highlighted the need for genome-scale analyses. Genomic sequencing technologies overcome many of these constraints by generating comprehensive genetic information that enables high-resolution characterization of pathogen populations. This transition from targeted detection to genomic surveillance represents a major advance in modern respiratory pathogen surveillance [46].

## 3. Genomic Surveillance: Expanding the Scope of Respiratory Pathogen Monitoring

The development of genomic sequencing has progressively expanded the resolution of respiratory pathogen surveillance, from analysis of selected genomic regions to comprehensive genome characterization and unbiased pathogen detection. These approaches can be selected according to the surveillance objective, sample characteristics, and available resources.

### 3.1. Whole-Genome Sequencing for High-Resolution Pathogen Characterization

Whole-genome sequencing (WGS) is a widely established approach for high-resolution characterization of respiratory pathogens, providing genome-wide information on genetic variation, lineage relationships, and pathogen evolution [14,20].

A representative example is SARS-CoV-2 genomic surveillance during the COVID-19 pandemic. Large-scale sequencing efforts allowed researchers to identify and track emerging viral lineages, including Alpha, Delta, and Omicron, through genomic mutations and phylogenetic relationships, supporting global monitoring of variant emergence [47,48].

Beyond viral surveillance, WGS is increasingly important for respiratory bacterial pathogens, particularly for antimicrobial resistance monitoring [21,23]. For example, genomic analysis of *Klebsiella pneumoniae*, *Acinetobacter baumannii*, and *Pseudomonas aeruginosa* has enabled identification of resistance-associated genes and their relationships with bacterial lineages and mobile genetic elements [49,50,51,52,53,54,55,56,57]. However, the presence of resistance-associated genes does not always directly predict phenotypic resistance, and genomic findings should therefore be interpreted alongside conventional antimicrobial susceptibility testing.

### 3.2. Targeted Sequencing for Focused Genomic Surveillance

Targeted sequencing extends molecular surveillance from pathogen detection to focused characterization of genetic variation by selectively enriching predefined genomic regions [45]. Amplicon sequencing and hybrid-capture sequencing are particularly useful when surveillance targets specific genetic features or requires high-throughput analysis of large numbers of specimens [58].

Respiratory syncytial virus (RSV) provides an important example of this application. The emergence of RSV vaccines and monoclonal antibodies has increased the need to monitor genetic variation in viral regions relevant to antigenicity and therapeutic susceptibility. An improved amplicon-based RSV sequencing assay was recently evaluated using 1071 clinical specimens collected during 2019–2023. The approach achieved >90% genome coverage at a minimum read depth of 20 in 96.83% of the 1071 clinical specimens tested, demonstrating the feasibility of high-throughput genomic surveillance directly from respiratory clinical samples.

Targeted sequencing has also been applied to respiratory viruses with more limited genomic surveillance resources. For human parainfluenza viruses (hPIV), an amplicon-based sequencing protocol developed for hPIV-1 and hPIV-2 generated 41 near-complete hPIV-1 genomes and 13 near-complete hPIV-2 genomes directly from clinical specimens. Phylogenetic analysis identified distinct circulating clusters, illustrating the utility of targeted sequencing for respiratory pathogens with limited genomic characterization [59].

Another important application of genomic surveillance is outbreak investigation. During an RSV-B outbreak among adult hematological patients at Hannover Medical School in Germany, target-capture sequencing was used to reconstruct RSV genomes directly from clinical specimens. The four outbreak-associated RSV-B genomes were identical to each other and clearly distinct from contemporaneously circulating RSV-B control strains, providing molecular evidence that supported the epidemiological investigation. Importantly, this study illustrates how genomic information can complement routine PCR-based screening and infection-control measures rather than replace them, highlighting the value of integrating conventional epidemiology with genomic analysis for outbreak investigation and control [60]. Overall, targeted sequencing offers a practical balance between genomic resolution and surveillance efficiency [43,61,62,63].

### 3.3. Metagenomic Next-Generation Sequencing for Unbiased Detection of Emerging Pathogens

Unlike targeted sequencing approaches, metagenomic next-generation sequencing (mNGS) enables pathogen detection without requiring predefined genomic targets [64]. By sequencing nucleic acids directly from clinical specimens, mNGS can simultaneously survey viral, bacterial, fungal, and other microbial sequences within a single sample. This unbiased strategy is particularly valuable when the etiological agent is unknown, when conventional targeted assays fail to identify a pathogen, or when mixed infections are suspected [40]. Its value for emerging infections was exemplified during the emergence of SARS-CoV-2, when metagenomic analysis of respiratory specimens contributed to the identification and genomic characterization of the previously unrecognized coronavirus.

A major challenge for respiratory mNGS is the high background of host and commensal nucleic acids in clinical specimens, which can substantially reduce the proportion of pathogen-derived reads and complicate downstream interpretation [65]. This limitation has prompted the development of host nucleic acid depletion, microbial enrichment, optimized sequencing depth, and improved bioinformatic filtering strategies. Studies of severe pneumonia have shown that combining host-depletion strategies with optimized sequencing and computational workflows can increase the effective microbial sequencing yield and improve the detection of low-abundance microorganisms [66]. These developments indicate that the clinical utility of mNGS increasingly depends not only on sequencing depth, but also on effective strategies for enriching pathogen signals and distinguishing clinically relevant findings from background microbial sequences. However, host depletion is not universally beneficial, as non-specific removal of microbial nucleic acids may also reduce the recovery of certain pathogens. The analytical performance of respiratory mNGS therefore depends on the balance between reducing host background and preserving microbial signals, rather than on sequencing depth alone.

In addition, detection of microbial nucleic acids by mNGS does not necessarily establish a causal relationship with the clinical condition, particularly in respiratory specimens where colonization and contamination may occur. Interpretation of mNGS results therefore requires integration of specimen type, microbial abundance or read metrics, clinical manifestations, and conventional microbiological evidence when available. In this context, mNGS should be viewed as a broad pathogen-detection approach that expands the range of detectable microorganisms, while clinical attribution remains dependent on integrated interpretation of genomic and clinical evidence.

### 3.4. Emerging Sequencing Technologies for Real-Time and Decentralized Surveillance

Recent advances in sequencing technologies have expanded the operational flexibility of genomic surveillance beyond conventional centralized sequencing laboratories. Oxford Nanopore sequencing represents one of the most promising platforms because of its portability, rapid sequencing capability, and potential for real-time genomic analysis [38].

During infectious disease outbreaks, portable sequencing platforms have demonstrated the ability to generate genomic information directly at or near the sampling site. For example, nanopore sequencing has been used during Ebola outbreaks in field settings to demonstrate the feasibility of genomic data generation outside centralized laboratories [67]. Similar approaches have also been explored for respiratory pathogens, including SARS-CoV-2, where rapid sequencing workflows enabled near-real-time sequence analysis and mutation characterization [68,69].

Although nanopore sequencing still faces challenges related to accuracy, infrastructure, and data interpretation, its portability and potential for near-real-time genomic analysis make it a promising approach for decentralized respiratory pathogen surveillance [62,70].

## 4. Public Health Applications of Genomic Surveillance

The integration of genomic information into respiratory pathogen surveillance has expanded public health monitoring beyond case detection toward characterization of pathogen evolution, transmission, antimicrobial resistance, and population-level changes. When combined with epidemiological and clinical information, genomic data can provide higher-resolution evidence for outbreak investigation, risk assessment, vaccine policy, and infection prevention. Importantly, the value of genomic surveillance depends not only on sequencing capacity but also on how genomic information is interpreted and translated into public health action.

### 4.1. Evolutionary Monitoring and Early Warning of Emerging Threats

Continuous genomic monitoring enables public health systems to track changes in circulating respiratory pathogens and identify genetic patterns that may warrant further epidemiological or phenotypic investigation [6]. This is particularly relevant for rapidly evolving respiratory viruses, for which changes in lineage distribution or functionally important genomic regions may alter population-level risk [9,37].

Influenza surveillance provides a long-established example [7,8,10]. Global genomic surveillance is combined with antigenic and epidemiological data to characterize viral evolution and inform seasonal vaccine composition [11]. For RSV, genomic surveillance has also been used to characterize circulating lineage distributions and genetic clusters. In Minnesota, whole-genome sequencing of 575 RSV-positive clinical specimens collected during July 2023–February 2024 identified distinct RSV-A and RSV-B lineage distributions and 23 clusters of genetically identical genomes [71,72].

Together, these examples illustrate that genomic surveillance can provide an early-warning signal by identifying changes in pathogen populations that warrant further epidemiological, antigenic, or clinical assessment, rather than directly indicating increased transmissibility, virulence, immune escape, or population-level risk.

### 4.2. Genomic Epidemiology for Transmission Investigation

Genomic epidemiology provides a means of distinguishing genetically related infections from epidemiologically unrelated cases [4], thereby improving the resolution of respiratory outbreak investigations [14]. This is particularly relevant in healthcare settings, where several patients may test positive for the same respiratory pathogen during the same period without necessarily belonging to a single transmission chain.

RSV provides a clear example [72]. During an RSV-B outbreak in a hematology-oncology and stem-cell transplant unit, whole-genome sequencing identified an identical RSV-B strain among 11 patients and healthcare workers [4], while contemporaneous RSV-B strains from epidemiologically unrelated individuals were genetically distinct. Variation in the G gene alone was insufficient to adequately discriminate the outbreak strains, highlighting the additional resolution provided by whole-genome analysis [69].

These findings demonstrate how genomic information can complement conventional epidemiological investigation by determining whether apparently similar infections are likely to represent transmission events or independent introductions. Such evidence can support more targeted infection prevention measures rather than relying solely on broad outbreak-control interventions.

### 4.3. Antimicrobial Resistance Surveillance and Infection Management

Genomic surveillance is particularly valuable for respiratory bacterial pathogens because antimicrobial resistance reflects both phenotypic resistance and the dissemination of resistant lineages and mobile genetic elements. Conventional antimicrobial susceptibility testing remains essential for individual patient management, whereas genomic analysis can provide additional information on the genetic basis and population structure of resistance [21].

For *Klebsiella pneumoniae*, *Acinetobacter baumannii*, and *Pseudomonas aeruginosa*, WGS can identify resistance-associated determinants and characterize the distribution of resistant clones [49,50,51,52,53,54,55,57]. This can help distinguish resistance arising through independent acquisition of resistance mechanisms from resistance associated with the expansion and spread of particular lineages.

Pneumococcal surveillance further illustrates the interaction between genomic evolution and resistance [56]. An international WGS study of 3233 invasive *Streptococcus pneumoniae* isolates from six countries showed that post-vaccine pneumococcal populations were shaped by serotype replacement and expansion of particular lineages, with non-vaccine serotypes showing distinct genomic and antimicrobial resistance profiles [67]. These findings illustrate that genomic surveillance can complement serotype-based monitoring by linking population changes to specific bacterial lineages and resistance patterns.

Thus, genomic surveillance can complement phenotypic susceptibility testing by linking resistance determinants to bacterial lineages and transmission patterns [73,74]. This information can support outbreak investigation and infection-control planning, while phenotypic susceptibility testing remains essential for patient-level treatment decisions.

### 4.4. Guiding Vaccine Strategies and Public Health Responses

Genomic surveillance can contribute to vaccine and preventive strategies by providing population-level information on the genetic composition of circulating respiratory pathogens. However, genomic data alone cannot determine vaccine effectiveness or establish antigenic escape. Their value lies in integration with antigenic, epidemiological, immunological, and clinical evidence to assess whether circulating pathogens remain appropriately matched to existing preventive interventions [75].

Influenza surveillance illustrates how genomic information can be integrated with antigenic, epidemiological, and immunological evidence to inform vaccine policy. Within the WHO Global Influenza Surveillance and Response System (GISRS), genetic characterization of circulating viruses is combined with antigenic analyses, epidemiological and clinical surveillance, and human serological studies to assess the relationship between circulating viruses and currently available vaccine viruses [8]. These integrated assessments are conducted twice annually and contribute to recommendations for the composition of seasonal influenza vaccines [28].

A similar principle is increasingly relevant to RSV as new preventive products enter routine use. For RSV, the introduction of vaccines and long-acting monoclonal antibodies has created a need for surveillance systems capable of evaluating their population-level performance under real-world conditions [6]. In contrast to simply monitoring mutations, recent RSV studies have begun to combine genomic information with vaccine effectiveness and clinical outcomes [76]. For example, a multicenter observational study in the United States evaluated RSV vaccine effectiveness against hospitalization and emergency department encounters among adults aged ≥60 years during the 2023–2024 season [77], illustrating the type of real-world clinical evidence required to assess newly introduced RSV vaccines. Genomic surveillance can complement such effectiveness studies by describing the genetic composition of RSV infections occurring in vaccinated and unvaccinated populations. This may help determine whether differences in circulating viral populations are associated with observed variation in vaccine performance, although such associations require confirmation through epidemiological, immunological, and clinical studies.

## 5. Future Perspectives: Toward AI-Enhanced Intelligent Genomic Surveillance

Although genomic surveillance has expanded the resolution and scope of respiratory pathogen monitoring, the increasing volume and heterogeneity of genomic, epidemiological, and clinical data create new challenges for timely interpretation and decision-making. Artificial intelligence (AI) and machine learning (ML) may provide potential approaches for extracting complex patterns from these multidimensional datasets, prioritizing signals of potential epidemiological importance, and supporting risk assessment [78]. Importantly, most current applications remain at the research or validation stage rather than constituting established components of routine respiratory pathogen surveillance. Future development should therefore focus on integrating AI with existing laboratory, genomic, and epidemiological workflows while maintaining appropriate human oversight.

### 5.1. AI-Assisted Genomic Data Interpretation

The rapid expansion of pathogen sequencing has increased the need for analytical approaches that can efficiently interpret genetic variation and identify changes with potential biological or epidemiological significance. Rather than replacing established bioinformatic pipelines, AI-based methods may provide an additional analytical layer for prioritizing mutations, predicting phenotypic characteristics, and identifying complex genotype–phenotype relationships [41].

Influenza provides a representative example. A recent machine learning study developed a model to predict antigenic characteristics of human influenza A(H3N2) viruses using HA1 sequences and associated metadata. The model was trained using historical data from influenza seasons between 2003 and 2021 and evaluated its ability to predict hemagglutination-inhibition (HI) assay results in subsequent seasons. The model could distinguish antigenically different variants and identify HA1 sites associated with antigenic change, suggesting that machine learning may assist the interpretation of genetic changes that are difficult to evaluate from sequence data alone [79].

Similar approaches have been explored for bacterial antimicrobial resistance [80]. Machine learning models have been developed to infer resistance phenotypes from WGS-derived genomic features in Gram-negative bacteria, including *Klebsiella pneumoniae* and *Acinetobacter baumannii* [81,82,83,84]. More recently, a WGS-based model trained on 5239 *K. pneumoniae* strains from geographically and temporally diverse cohorts achieved high predictive performance across 11 antibiotics, illustrating the potential of AI-assisted genomic interpretation for expanding from resistance-gene detection toward genotype-based resistance prediction.

These studies illustrate how AI can potentially transform genomic data from descriptive information into prioritized predictions of phenotypic or epidemiological relevance [85,86]. Nevertheless, such predictions should currently be regarded as complementary evidence rather than replacements for antigenic assays, antimicrobial susceptibility testing, or expert genomic interpretation.

### 5.2. Predictive Epidemiology and Risk Assessment

A further potential application of AI is the integration of genomic surveillance with epidemiological and other population-level data to identify changes in transmission risk before conventional surveillance signals become fully apparent. This represents an important transition from retrospective genomic analysis toward predictive surveillance.

Recent work has demonstrated that machine learning can support early warning for respiratory disease activity even when genomic information is not yet incorporated directly. In a 2026 *Nature Communications* study, researchers developed a real-time machine learning system combining Google search trends with conventional epidemiological data to predict the onset and peak of respiratory disease outbreaks across all 50 U.S. states. The system simultaneously monitored influenza-like illness and hospitalizations associated with influenza and RSV, demonstrating the feasibility of using machine learning to integrate heterogeneous respiratory surveillance signals for earlier outbreak detection [87]. Importantly, this study did not incorporate pathogen genomic information and therefore should be considered evidence for AI-assisted epidemiological surveillance rather than direct evidence of AI-based genomic surveillance.

Building on this concept, future genomic surveillance frameworks could incorporate pathogen-derived features into predictive models. For example, lineage frequency, mutation profiles, genomic diversity, and transmission clusters could be combined with case counts, hospitalization data, geographic information, and environmental or behavioral indicators. Such integration may allow models to distinguish simple increases in pathogen detection from changes associated with specific lineages or transmission patterns.

However, predictive epidemiology should not be interpreted as direct prediction of future outbreaks from genomic data alone. Model performance is strongly influenced by sampling density, geographic representativeness, reporting delays, changes in testing practices, and the quality of linked epidemiological data. Therefore, future AI-based surveillance systems will require prospective validation across different pathogens, populations, and surveillance settings.

### 5.3. AI-Assisted Precision Infection Control

A potential future application of AI is to integrate genomic relatedness with patient, temporal, spatial, and healthcare-associated exposure information to prioritize transmission events for further investigation [76,78]. Genomic surveillance can identify clusters of genetically related respiratory pathogens, while epidemiological information can determine whether the affected individuals shared a ward, healthcare worker, or exposure period. An AI-assisted system could potentially integrate these signals to identify unusual transmission patterns and prioritize clusters for expert review.

This approach may be particularly useful in healthcare-associated respiratory infections, where surveillance resources are limited and not every genetically related case requires the same level of investigation. Rather than automatically generating intervention decisions, AI could function as a decision-support layer that facilitates integration of laboratory and epidemiological information and helps infection-control teams focus on clusters requiring further assessment [85,86].

Importantly, such applications remain prospective rather than established routine practice. AI-generated risk scores should therefore be interpreted alongside genomic evidence, clinical information, and epidemiological investigation rather than being used as autonomous determinants of isolation, treatment, or other infection-control measures.

### 5.4. Challenges and Future Perspectives

Despite the potential of AI-assisted genomic surveillance, several barriers must be addressed before these approaches can be incorporated reliably into routine respiratory pathogen surveillance.

First, data quality and representativeness remain fundamental limitations [78]. Machine learning models require sufficiently large and representative datasets linking genomic information with reliable phenotypic, epidemiological, or clinical outcomes. However, sequencing capacity and sampling strategies differ substantially between regions. Models trained using data from specific populations or surveillance systems may therefore perform poorly when applied to genetically or epidemiologically different populations.

Second, external validation and model interpretability are essential for public health applications [22]. High performance in retrospective datasets does not necessarily indicate reliable performance in prospective surveillance. Models must be evaluated using temporally and geographically independent datasets and should provide interpretable evidence regarding the genomic or epidemiological features contributing to their predictions. This is particularly important when model outputs may influence resource allocation or infection-control decisions.

Third, integration with existing surveillance infrastructure remains challenging. AI systems must operate alongside laboratory diagnostics, sequencing pipelines, genomic databases, epidemiological reporting systems, and public health response networks. The WHO’s recent guidance for integrated respiratory surveillance illustrates the broader direction of surveillance development: specimen collection, testing, sequencing, data sharing, analysis, and public health action should function as connected components rather than isolated activities [35].

Finally, AI should currently be regarded as an augmentation technology rather than an autonomous surveillance system. The most realistic near-term model is likely to involve AI assisting experts with data integration, signal prioritization, prediction, and decision support, while laboratory confirmation, epidemiological interpretation, and public health intervention remain subject to established scientific and institutional processes.

Collectively, these considerations suggest that the future of respiratory pathogen surveillance will depend not simply on developing more powerful AI algorithms, but on building an integrated framework in which molecular diagnostics, sequencing, epidemiology, clinical data, and computational intelligence can operate together. The transition toward intelligent genomic surveillance should therefore be viewed as an incremental process of data integration, model validation, prospective evaluation, and controlled implementation, rather than a replacement of existing surveillance technologies (Table 2).

## 6. Conclusions

Respiratory pathogens continue to pose major challenges to global public health, reflecting their genetic diversity, dynamic transmission patterns, and, for bacterial pathogens, the emergence and spread of antimicrobial resistance. Conventional molecular diagnostics remain indispensable for rapid and scalable pathogen detection, whereas genomic approaches provide additional resolution for characterizing pathogen evolution, transmission, and resistance. These approaches should therefore not be considered competing technologies. Instead, their complementary capabilities can be integrated to address different surveillance objectives across clinical diagnosis, outbreak investigation, pathogen characterization, and public health response.

The central challenge for future respiratory pathogen surveillance is therefore not simply to adopt increasingly advanced sequencing technologies, but to determine how different diagnostic and genomic approaches can be appropriately combined according to surveillance objectives, pathogen characteristics, clinical context, and available resources. An integrated framework linking molecular detection, targeted or whole-genome sequencing, metagenomic analysis, epidemiological investigation, and clinical information can provide a more comprehensive basis for interpreting respiratory pathogen activity. This perspective is consistent with current international efforts to strengthen integrated respiratory pathogen surveillance and end-to-end genomic surveillance.

Looking forward, artificial intelligence may provide an additional layer for integrating these heterogeneous data sources and assisting genomic interpretation, risk assessment, and surveillance prioritization. However, current AI applications remain largely at the research and validation stage, and their translation into routine public health practice will depend on data quality, model generalizability, external validation, interpretability, infrastructure, and appropriate human oversight. AI should therefore be viewed as an augmentation of existing surveillance systems rather than an autonomous replacement for laboratory testing, genomic analysis, or expert decision-making. Ultimately, the development of respiratory pathogen surveillance should move from a technology-centered model toward an integrated and objective-oriented framework in which different methods are selected and combined according to the information required for a specific public health question. Such a framework may enable surveillance systems to characterize their evolution and transmission, identifying signals that warrant further risk assessment, and supporting targeted interventions. Continued methodological validation, standardized data infrastructures, and international collaboration will be essential for translating this integrated approach into sustainable and actionable respiratory pathogen surveillance.

## Figures and Tables

**Table 1:** Evolution of respiratory pathogen surveillance technologies and their surveillance capabilities.

Surveillance Approach	Representative Technologies	Primary Capability	Typical Applications	Major Strengths	Major Limitations	References
Phenotypic surveillance	Culture-based methods; antigen detection	Pathogen isolation and phenotypic characterization	Clinical diagnosis; antimicrobial susceptibility testing (AST); isolate preservation	Enables recovery of viable organisms and direct phenotypic characterization	Time-consuming; requires viable pathogens; limited applicability for some respiratory viruses	[36]
Targeted molecular surveillance	PCR; RT-qPCR	Rapid detection of predefined respiratory pathogens	Routine diagnosis; outbreak screening; laboratory confirmation	High analytical sensitivity and specificity; rapid turnaround; readily scalable	Requires predefined targets; provides limited information on pathogen evolution and transmission dynamics	[37]
Multiplex molecular surveillance	Multiplex PCR respiratory panels	Simultaneous detection of multiple predefined pathogens	Syndromic diagnosis; co-infection detection; clinical decision support	Broadens pathogen coverage while maintaining rapid detection	Restricted to predefined targets; limited information on pathogen evolution and genomic relationships	[7]
Targeted genomic surveillance	Amplicon sequencing; hybrid-capture sequencing	Characterization of predefined genomic regions or genetic markers	Lineage assignment; mutation monitoring; focused genomic surveillance; outbreak investigation	Higher genomic resolution than molecular detection; suitable for high-throughput analysis of selected targets	Dependent on predefined genomic regions; changes outside targeted regions may be missed	[38]
Whole-genome surveillance	Whole-genome sequencing (WGS); short- and long-read sequencing platforms	Comprehensive genomic characterization of known pathogens	Variant and lineage monitoring; genomic epidemiology; transmission investigation; antimicrobial resistance surveillance	Provides genome-wide information for evolutionary analysis, pathogen differentiation, and resistance characterization	Requires sequencing infrastructure, bioinformatics expertise, and standardized analytical workflows	[39]
Metagenomic surveillance	Shotgun metagenomic next-generation sequencing (mNGS)	Unbiased detection and characterization of microbial sequences	Investigation of unexplained or severe pneumonia; detection of unexpected pathogens; mixed-infection analysis	Enables detection of unexpected pathogens without predefined targets and can simultaneously characterize multiple microbial agents	Host nucleic acid background; complex interpretation; relatively high cost; clinical significance of detected organisms may require additional validation	[40]
Intelligent genomic surveillance	Real-time sequencing; AI-assisted genomic analysis; integrated surveillance platforms	Integration of genomic, epidemiological, and clinical data for surveillance enhancement	Early warning; outbreak risk assessment; predictive surveillance; precision infection control	Integrates genomic, epidemiological, and clinical information to support more timely and potentially proactive surveillance	Dependent on data quality, computational infrastructure, interoperability, model validation, and appropriate clinical/public health implementation	[41]

**Table 2:** Emerging trends and future priorities for intelligent genomic surveillance of respiratory pathogens.

Emerging Trend	Current Limitation	Research Focus	Potential Public Health Value
AI-assisted genomic interpretation	Limited scalability and efficiency of manual genomic analysis	Model validation, explainable AI, and generalizable algorithms	Faster identification of variants, mutations, and AMR determinants
Predictive epidemiology	Limited ability to anticipate pathogen emergence and outbreak dynamics	Integration of genomic, epidemiological, and clinical data for predictive modeling	Earlier risk assessment and improved outbreak preparedness
Real-time genomic surveillance	Delays in sequencing, analysis, and data reporting	Portable sequencing, automated bioinformatic pipelines, and cloud-based analysis	Timelier outbreak detection and public health response
Precision infection control	Infection control strategies remain largely generalized	Risk stratification based on integrated genomic and epidemiological evidence	More targeted infection prevention and optimized resource allocation
Global genomic surveillance	Uneven sequencing capacity, fragmented data sharing, and inconsistent standards	International collaboration, standardized protocols, and interoperable surveillance platforms	Improved global preparedness and coordinated responses to emerging respiratory pathogens

## Data Availability

No new data were created or analyzed in this study.

## Abbreviations

The following abbreviations are used in this manuscript:

AI	Artificial Intelligence
AMR	Antimicrobial Resistance
FluNet	Influenza Network
mNGS	Metagenomic Next-Generation Sequencing
PCR	Polymerase Chain Reaction
qPCR	Quantitative Polymerase Chain Reaction
RespiMart	Respiratory Virus Mart
RSV	Respiratory Syncytial Virus
RT-qPCR	Reverse Transcription Quantitative Polymerase Chain Reaction
SARS-CoV-2	Severe Acute Respiratory Syndrome Coronavirus 2
WGS	Whole-Genome Sequencing
WHO	World Health Organization
